# Supplementary figures and images for: Functional Characterization of LTR12C as Regulators of Germ-Cell-Associated TA-p63 in U87-MG and T98-G In Vitro Models
Source: Cells. 2025 Jun 5;14(11):852. doi: 10.3390/cells14110852 (PMC12154421; doi:10.3390/cells14110852)

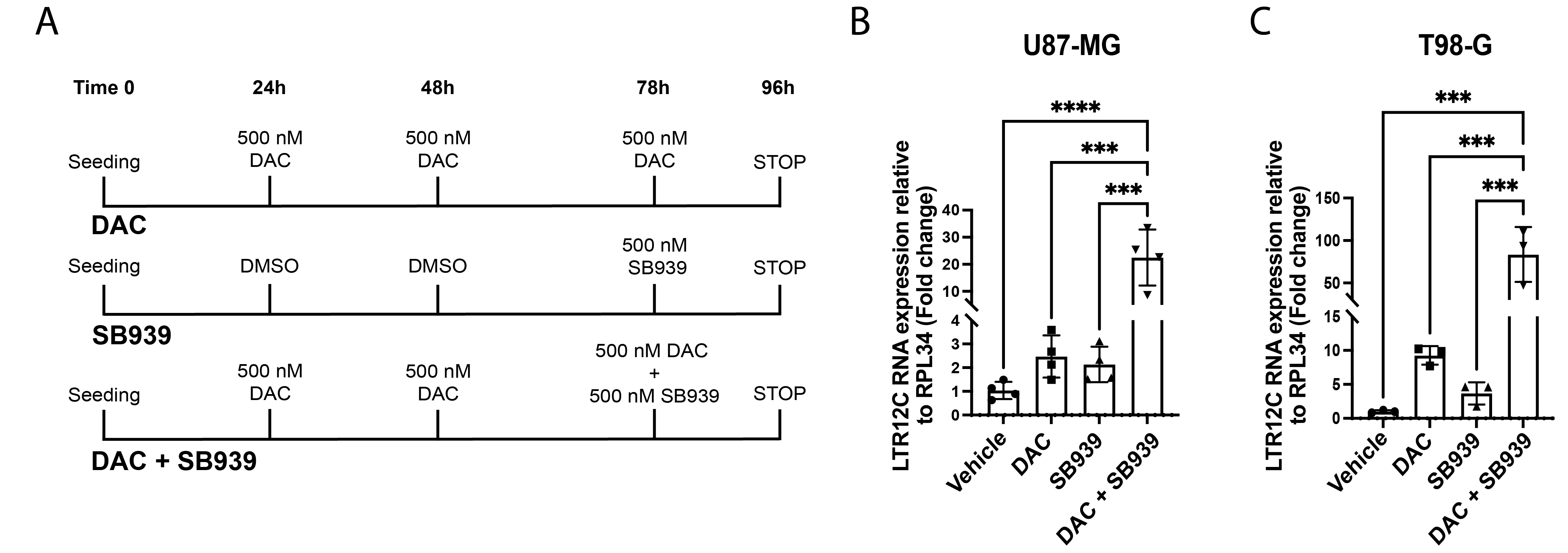

Supplement: Supplementary file 1 [file cells-14-00852-s001.zip › Figure S1.png]

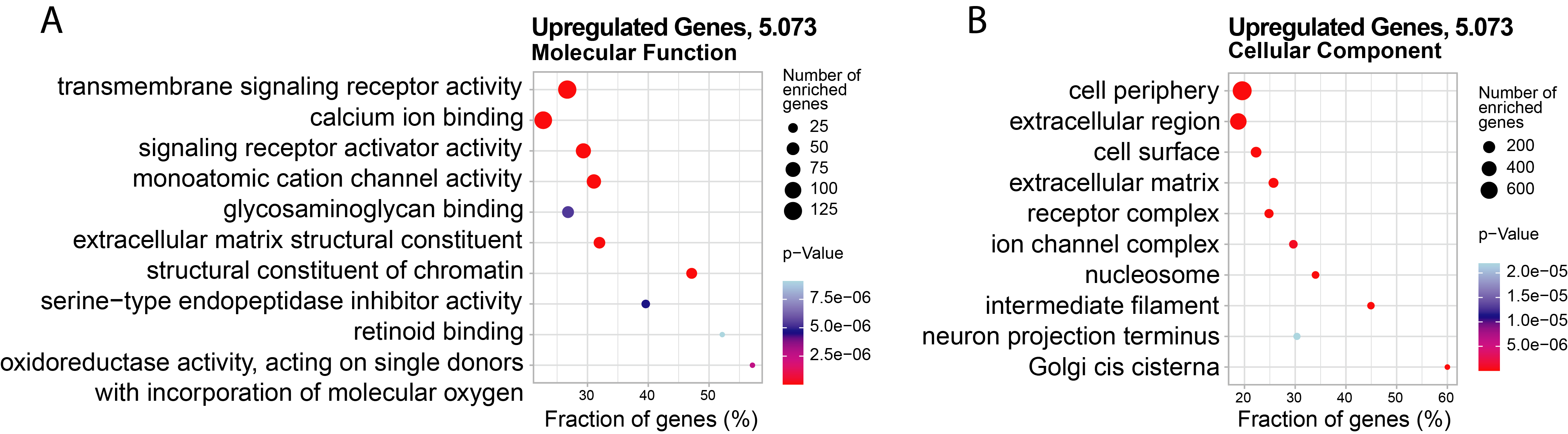

Supplement: Supplementary file 1 [file cells-14-00852-s001.zip › Figure S2.png]

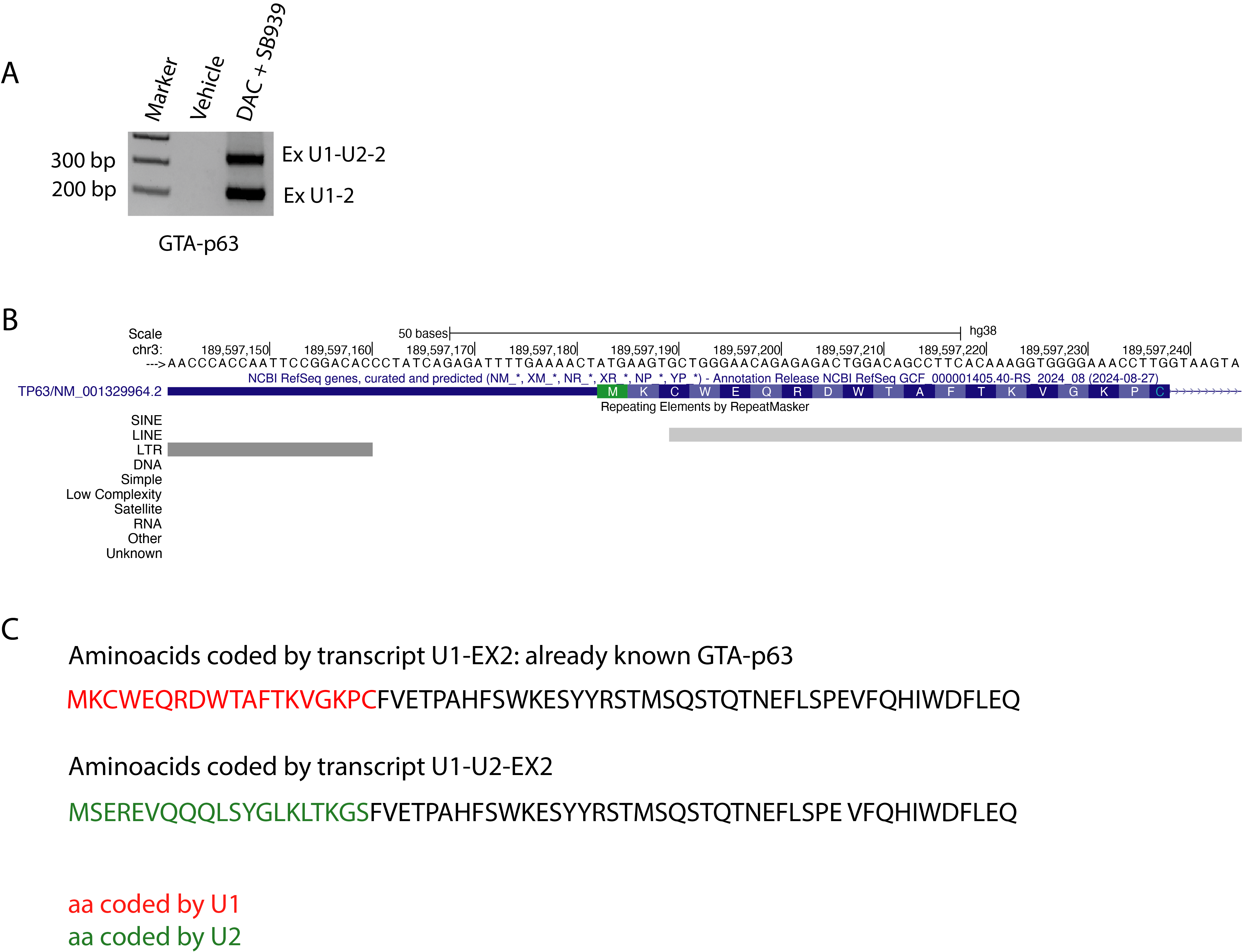

Supplement: Supplementary file 1 [file cells-14-00852-s001.zip › Figure S3.png]
